# Patterns for Child Protective Service Referrals in a Pediatric Burn Cohort

**DOI:** 10.7759/cureus.51525

**Published:** 2024-01-02

**Authors:** Sima Vazquez, Ankita Das, Eris Spirollari, Jose Dominguez, Kerri Finnan, Joseph Turkowski, Irim Salik

**Affiliations:** 1 Department of Neurological Surgery, New York Medical College, Valhalla, USA; 2 Department of Neurosurgery, Westchester Medical Center, Valhalla, USA; 3 Burn Center, Department of Surgery, Westchester Medical Center, Valhalla, USA; 4 Department of Anesthesia, Westchester Medical Center, Valhalla, USA

**Keywords:** trauma pediatric, child abuse and neglect, referral pathway, child safety, burn injury

## Abstract

Background: Pediatric non-accidental trauma often necessitates the involvement of protective services. However, the subjectivity and lack of standardization of referral infrastructure may result in some discrepancies between referral patterns and instances of child abuse.

Methods: An institutional retrospective chart review was conducted between 2015 and 2021, in which all cases of patients under the age of 14 who suffered a burn injury and received a child protective service (CPS) consult were reviewed. Baseline demographics and characteristics were defined. Multivariate analysis was utilized to identify predictors of CPS involvement, while the regression analysis was employed to parse associations between burn injuries and CPS involvement.

Results: Between July 2015 and December 2021, 340 patients (median age two years, IQR: 1-6 years) under the age of 14 who experienced a burn injury were evaluated. Forty-four (12.9%) of the patients’ cases received a CPS referral, of which three (0.9%) resulted in a CPS intervention. The most common mechanism of burn within the cohort was scald (241 patients, 70.9%). The median total body surface area (TBSA) was 3.0% (IQR: 1.0%-6.0%), and 76 (22.4%) suffered a high TBSA (>75^th^ percentile). Caucasian race (p < 0.001) and scald mechanisms (p = 0.014) were associated with higher TBSA. When considering how such injuries translated to CPS referrals, increasing age was found to be associated with a decreased likelihood of CPS involvement. Meanwhile, the Black race (p = 0.027) and increasing area deprivation index (ADI) (p = 0.038) were associated with CPS involvement. Those with CPS involvement experienced a greater length of hospital stay (p = 0.001). Black race and intensive care unit level of care were found to be positive predictors of CPS involvement. In total, three (6.82%) of the 44 cases with CPS involvement were found to be substantiated. The three children who required CPS intervention were discharged to foster care settings.

Conclusion: Hospitalized pediatric burn injuries must be investigated due to concern of child abuse, yet external factors such as race and socioeconomic status may play a role in the involvement of CPS. Such referrals may not always be substantiated and could lead to further injurious sequelae for children and their families.

## Introduction

Child abuse and neglect are common mechanisms of injury in pediatric burn patients [1-6]. The literature has previously shown the children at the highest risk for abuse are under three years of age, admitted with scald burns, and from a single-parent household [1-6]. Although some objective tools exist, a large component of the detection of non-accidental trauma remains subjective, as there are no universal child abuse management pathways or gold standards for evaluation [7,8]. Protective service agencies provide valuable intervention to protect children and promote their well-being in the appropriate contexts. However, one study comparing referrals from emergency department (ED) providers, skeletal survey results, and recommendations from child abuse experts showed that child protective service (CPS) reporting diverged from expert’s recommendations, and CPS was called frequently by ED providers in cases that experts deemed as having an indeterminate likelihood of abuse [8]. Another study showed factors of socio-demographic status, parental characteristics, history of contact with authorities, physical findings, and suspicious behaviors of children and parents to be associated with CPS or police referrals [7]. According to the U.S. Department of Health & Human Services, only 17.7% of reported cases in 2013 were substantiated upon investigation [9]. Substantiation has been associated with the severity of harm, parent risk factors, housing risk factors, police referrals, and involvement of multiple forms of maltreatment [10]. The discrepancy between referral and substantiation may be due to over-referral or under-investigation, both of which have considerable implications for further damage to the child and family [11]. As referrals rely on suspicion of abuse, over-reporting is inherently likely, yet we aim to investigate if certain demographic characteristics are associated with a greater likelihood of CPS referrals. This single-center retrospective review investigates a pediatric burn cohort to elucidate risk factors for CPS referrals and characteristics associated with case substantiation.

## Materials and methods

Data source and patient selection

With IRB approval, in this retrospective cohort study, all patients under the age of 14 who were hospitalized at our institution for burn injury and received a CPS consult between July 1, 2015, and December 31, 2021, were reviewed. All patients were included, and no specific exclusion was applied as each case was examined individually. Demographic variables of age, sex, race, ethnicity, and home address were collected. Mechanism of burn, treatments received, total body surface area (TBSA) involved, CPS intervention, and hospital course were also collected.

Data characteristics and outcomes measured

Patient demographics and characteristics, including age, race, ethnicity, and insurance status, were described. Area deprivation index (ADI) was calculated using the Neighborhood Atlas tool [12]. Higher ADI signifies a higher level of deprivation. The mechanism of burn injury was collected, along with burn severity, defined using the TBSA of burn involvement. Intensive care unit (ICU) requirements and hospital length of stay (LOS) were also analyzed. High TBSA was defined as TBSA greater than the 75th percentile or 6.0%. CPS involvement and type of intervention were also described. CPS involvement was defined as a referral that resulted in a CPS investigation of the patient’s case. CPS intervention constitutes removal of the child from his or her current living situation and discharge to foster care.

Statistical analysis

Categorical variables were compared using Pearson’s chi-squared test. Continuous variables were evaluated using univariate logistic or linear regression. Multivariate regression analysis was utilized to explore the predictors of CPS. Statistical Product and Service Solutions (SPSS) was used for analysis, and statistical significance was set at < 0.05 (released 2020; version 28.0; IBM SPSS Statistics for Windows, IBM Corp, Armonk, NY).

## Results

Baseline demographics and characteristics

A total of 340 patients under the age of 14 who had suffered a burn were identified, of which 44 (12.9%) had CPS involvement and three (0.9%) had CPS intervention. Median age was two (IQR: 1-6) years old, and 165 (48.5%) patients were female. A total of 165 (48.5%) patients identified as Caucasian, 70 (20.6%) identified as Black, and 11 (3.2%) patients identified as Asian. Meanwhile, 161 (47.4%) patients were on Medicaid insurance, 161 (47.4%) patients had private insurance, and 16 (4.7%) patients had other types of insurance. The median ADI was 24 (IQR: 12-50).

The most common mechanism of burn was scald (241, 70.9%), followed by contact (50, 14.7%), flame (30, 8.8%), electric (6, 1.8%), and friction (5, 1.5%). Median TBSA was 3.0% (IQR: 1.0%-6.0%), and 76 (22.4%) patients suffered high TBSA. ICU stay was required for 46 (13.5%) patients. Median LOS was five days with an IQR of two to eight days (Table 1). Caucasian race (p < 0.001) and scald mechanism (p = 0.014) were associated with high TBSA (Table 2).

**Table 1:** Baseline Characteristics of the Cohort. Abbreviations: IQR - interquartile range; ADI - area deprivation index; TBSA - total body surface area; ICU - intensive care unit; LOS - length of stay; CPS - child protective services

Parameter	Values (n=340)
Demographics	
Age (Median, IQR), years	2 (1-6)
Female	165 (48.5%)
Caucasian	175 (51.5%)
Black	70 (20.6%)
Asian	11 (3.2%)
Other Race	83 (24.5%)
Medicaid	161 (47.4%)
Private	161 (47.4%)
Other Insurance	16 (4.7%)
ADI (Median, IQR)	24 (12-50)
Mechanisms	
Scald	241 (70.9%)
Contact	50 (14.7%)
Flame	30 (8.8%)
Electric	6 (1.8%)
Friction	5 (1.5%)
Severity	
TBSA (Median, IQR)	3.0% (1.0%-6.0%)
High TBSA (>6.0%)	76 (22.4%)
ICU	46 (13.5%)
LOS (Median, IQR), days	5.0 (2.0-8.0)
CPS Involvement	
CPS Called	44 (12.9%)
CPS Intervention	3 (0.9%)

**Table 2:** Factors Associated with Severity.

	Low TBSA (263, 77.6%)	High TBSA (76, 22.4%)	OR (95%CI)	P value
Demographics				
Age				
Female	124 (47.1%)	41 (53.9%)	1.313 (0.787-2.191)	0.301
Caucasian	121 (46%)	54 (71.1%)	2.881 (1.659-5.002)	< 0.001
Black	59 (22.4%)	11 (14.5%)	0.585 (0.29-1.18)	0.149
Asian	10 (3.8%)	1 (1.3%)	0.337 (0.042-2.678)	0.467
Other Race	73 (27.8%)	10 (13.2%)	0.394 (0.192-0.808)	0.01
Medicaid	120 (45.6%)	41 (53.9%)	1.396 (0.836-2.33)	0.241
Private	130 (49.4%)	31 (40.8%)	0.705 (0.42-1.182)	0.195
Other Insurance	12 (4.6%)	4 (5.3%)	1.162 (0.364-3.712)	1
ADI	59 (22.3%)	23 (30.3%)	1.508 (0.854-2.663)	0.172
Mechanism				
Scald	178 (67.7%)	63 (82.9%)	2.314 (1.207-4.435)	0.014
Contact	48 (18.3%)	2 (2.6%)	0.121 (0.029-0.51)	< 0.001
Electric	6 (2.3%)	0 (0%)	0.977 (0.959-0.995)	0.344
Flame	20 (7.6%)	10 (13.2%)	1.841 (0.822-4.123)	0.167
Friction	5 (1.9%)	0 (0%)	0.981 (0.965-0.998)	0.355

Associations with CPS involvement

Increasing age was associated with a decreased likelihood of CPS involvement (p = 0.009). Black race (p = 0.027) and increasing ADI (p = 0.038) were associated with CPS involvement (Table 3). There were no associations with mechanisms of injury or TBSA and CPS involvement. The CPS involvement cohort had increased LOS (p = 0.001) and increased likelihood of ICU requirement (p = 0.002) (Table 3).

**Table 3:** Factors Associated with CPS Involvement. *One patient did not have CPS data (295+44=399). Scald associated with high TBSA - (OR: 2.314 (95% CI: 1.207-4.435) p = 0.014).

	Not CPS (295, 86.8%)	CPS (44, 12.9%)	OR (95%CI)	P value
Demographics				
Age			0.859 (0.766-0.963)	0.009
Female	144 (48.8%)	21 (47.7%)	0.957 (0.508-1.805)	1.00
Caucasian	158 (53.6%)	17 (38.6%)	0.546 (0.285-1.044)	0.076
Black	55 (18.6%)	15 (34.1%)	2.257 (1.133-4.494)	0.027
Asian	9 (3.1%)	2 (4.5%)	1.513 (0.316-7.245)	0.641
Other Race	73 (24.7%)	10 (22.7%)	0.894 (0.421-1.899)	0.853
Medicaid	141 (47.8%)	20 (45.5%)	0.91 (0.482-1.719)	0.872
Private	139 (47.1%)	22 (50%)	1.122 (0.596-2.115)	0.748
Other Insurance	14 (4.7%)	2 (4.5%)	0.956 (0.21-4.355)	1.00
ADI			1.103 (1.001-1.025)	0.038
Mechanism				
Scald	213 (72.2%)	28 (63.6%)	0.674 (0.346-1.31)	0.285
Contact	41 (13.9%)	9 (20.5%)	1.593 (0.713-3.557)	0.257
Electric	5 (1.7%)	1 (2.3%)	1.349 (0.154-11.823)	1
Flame	24 (8.1%)	6 (13.6%)	1.783 (0.685-4.642)	0.252
Friction	5 (1.7%)	0 (0%)	0.983 (0.968-0.998)	0.624
Severity				
High TBSA	62 (21%)	14 (31.8%)	1.754 (0.877-3.509)	0.122
LOS			1.076 (1.029-1.124)	0.001
ICU	33 (11.2%)	13 (29.5%)	3.329 (1.585-6.992)	0.002

Predictors of CPS involvement

On multivariate analysis, increasing age was a negative predictor of CPS involvement (p = 0.015), while Black race (p = 0.009) and ICU requirement (p = 0.006) were positive predictors of CPS involvement (Table 4).

**Table 4:** Multivariate Regression for CPS.

	OR (95%CI)	P value
ADI	1.008 (0.995-1.021)	0.244
Age	0.868 (0.775-0.073)	0.015
Black	2.766 (1.284-5.957)	0.009
Female	1.073 (0.547-2.103)	0.838
Medicaid	0.743 (0.373-1.480)	0.398
TBSA	1.167 (0.489-2.786)	0.727
ICU	3.63 (1.44-9.151)	0.006

Characteristics of patients with CPS intervention

Of the 44 cases with CPS involvement, three (6.82%) cases were substantiated with CPS intervention. One case was a Black two-year-old female with an ADI of 23 and private insurance. The mechanism of injury was scald involving bilateral feet, covering 2.0% TBSA. Her hospital LOS was six days, and she was discharged to foster care via CPS intervention. The second case was a 13-year-old female of other race. She was privately insured with a calculated ADI of 24. She suffered a contact burn involving her left hand, covering 1.0% TBSA. Her LOS was 10 days, and she was discharged to foster care. The last case was a two-year-old black male with a calculated ADI of 75 on Medicaid insurance. He suffered a contact burn involving his left leg and covering 2.0% TBSA. He was hospitalized for three days before being discharged to foster care. None of the three patients required ICU level of care (Table 5).

**Table 5:** Characteristics of Patients with CPS Intervention. *Every CPS call was cleared except for the following three.

	Age (Sex)	Race	ADI	Insurance	Mechanism of Injury, Location	TBSA, %	LOS, days	Type of Intervention
1	2 (F)	Black	23	Private	Scald, B/l Feet	2.0%	6	Discharge to foster care
2	13 (F)	Other	24	Private	Contact, L hand	1.0%	10	Discharge to foster care
3	2 (M)	Black	75	Medicaid	Contact, L leg	2.0%	3	Discharge to foster care

## Discussion

Socioeconomic factors were significantly associated with CPS involvement in our pediatric burn population. Our institutional study found associations between CPS involvement and both black race and high ADI. One study of children in California shows the importance of distinguishing between race and socioeconomic status [13]. Putnam-Hornstein et al. showed that the higher rate of CPS referrals and substantiations among black children compared to white children reversed when controlling for socioeconomic factors, such as the use of public insurance [13]. A systematic review also found an increased incidence of pediatric burns in patients from low-income households, deprived areas, rented accommodations, or single-parent families [14]. These studies highlight the complex relationships between race, socioeconomic status, and biases in the healthcare system that must be considered when assessing NAT. Development of a multidisciplinary, standardized approach to evaluation and intervention for patients suspected of NAT is warranted.

Multiple studies throughout the United States and Canada demonstrate that CPS investigations and substantiated cases are more prevalent in black patients [13-18]. One study of Ontario’s child welfare system showed that investigations involving white families doubled, while those involving black families quadrupled between 1998 and 2003 [15]. Many factors may contribute to the disproportion of investigation between races, including systemic or provider biases, along with socioeconomic disparities associated with race. Of the patients discharged to foster care in our cohort, two (66.7%) were of black race, while one was of ‘other’ race. Two of the three patients lived in zip codes associated with ADI scores lower than the national 25th percentile, while only one patient lived in an area with high ADI. Further studies are warranted to parse out the effects of race/ethnicity, financial stability, and geographic location on child maltreatment.

The requirement of ICU-level care was the strongest predictor of CPS involvement. In our study, ICU-level care serves as a marker of overall injury severity. Patients may have required ICU admission due to a multitude of factors, including polytrauma, location or mechanism of burn, burn depth, or burn TBSA. Studies show an association between NAT and mortality [19,20]. Severe injury in a child may easily raise suspicion for NAT, but studies analyzing both suspected and substantiated cases suggest that the mechanism of injury, the presence of witnesses and history corroboration, and additional injuries are more specific identifiers of abuse than injury severity itself [8,19]. In our cohort, none of the three patients discharged to foster care required ICU level of care.

Healthcare resource utilization was increased in the CPS involvement cohort. In concordance with our findings, prior research has shown cases with CPS involvement have increased hospital LOS and costs [21,22]. One study shows an additional cost of $6,521.93 US dollars after medical clearance for discharge due to continued hospitalization for CPS-related factors [21]. Furthermore, reports of increased ICU stay after controlling for injury severity in CPS cases have been described [23]. These findings suggest that cases associated with CPS investigations independently contribute to a significant increase in the healthcare resource burden, an important consideration given the low rate of substantiation [24]. While we are not suggesting that suspicion of NAT should be ignored or minimized by healthcare practitioners, we conclude there should be a standardized system in place to assess for maltreatment, one that is less reliant on providers or systemic biases.

In addition to unfavorable inpatient hospitalization courses, contact with CPS is associated with worsened child outcomes [25-27]. Additively, minority groups may have fewer social safety nets, a lower threshold for maltreatment by child welfare groups, and child welfare/healthcare worker implicit bias that may impact CPS referral dispositions. Improved child abuse pathways and clear indications for CPS referrals are necessary - one study showed improvement in substantiation rate (up to 53.5%) with a program involving collaboration between established hospital child protection teams and child protection officers [11]. After gathering data from a large literature review, Maguire et al. outlined a screening tool to categorize burn injuries into one of three categories: intentional scald must be excluded, intentional scald should be considered, and intentional scald unlikely [28]. The tool focuses on the mechanism and pattern of burn injury, the presence of associated or co-existing injuries, and historical or social factors [28]. Manan et al. also proposed a multi-specialty approach and practice points for history-taking in cases of suspected abuse [27]. Healthcare providers should learn to recognize discrepancies, identify uncooperative caregiver behavior, and conduct independent interviews with the child [27]. Further investigation to develop multi-disciplinary support for patients undergoing CPS investigation is warranted.

Limitations

Although our conclusions are in line with previous data on the overrepresentation of minority populations in CPS referrals, our study has several limitations. Our six-year retrospective review is prone to missing data points, including additional injuries, comorbidities, or history of previous contact with authorities. We could not find a documented reason for a CPS call in each case, leaving us unaware of the healthcare provider’s decision-making process in activating CPS. In the future, a qualitative study of provider decision-making in these instances may shed light on their methodologies and personal algorithms for initiating referrals. Long-term follow-up was unavailable, and ongoing CPS investigations were considered resolved when the child was deemed safe to return home. Additionally, with a small sample size, it is possible to overestimate the differences between our comparison groups; therefore, future multi-center research is encouraged. Lastly, this is a focused study of our institutional findings; we acknowledge that our population and the demographic findings of the burn cohort may differ across the nation.

## Conclusions

Our analysis found that Black race and increased ADI were associated with CPS referral, leading to prolonged hospital LOS and cost. Income poverty and its correlates remain fertile ground for the advancement of healthcare equity agendas. As stewards of safety for our vulnerable pediatric population, we must assess racial and socioeconomic disparities as related to individual providers, along with barriers to equitable healthcare and social advancement. Further research should be conducted to analyze whether racial disparities in CPS referrals arise from factors specific to minorities, systemic inequities present in the management of child abuse allegations, or a complex interplay of the two.
